# Detecting gastrointestinal manifestations in patients with systemic sclerosis using anti-gAChR antibodies

**DOI:** 10.1186/s13075-020-2128-z

**Published:** 2020-02-21

**Authors:** Shunya Nakane, Masataka Umeda, Shin-ya Kawashiri, Akihiro Mukaino, Kunihiro Ichinose, Osamu Higuchi, Yasuhiro Maeda, Hideki Nakamura, Hidenori Matsuo, Atsushi Kawakami

**Affiliations:** 1 Department of Neuroimmunology, Nagasaki University Graduate School of Biomedical Sciences, Nagasaki, Japan; 2 Department of Clinical Research, Nagasaki Kawatana Medical Center, Nagasaki, Japan; 3 Department of Neurology, Nagasaki Kawatana Medical Center, Nagasaki, Japan; 4 Department of Molecular Neurology and Therapeutics, Kumamoto University Hospital, 1-1-1, Honjo, Chuouku, Kumamoto-shi, Kumamoto, 860-8556 Japan; 5 Department of Immunology and Rheumatology, Unit of Translational Medicine, Graduate School of Biomedical Sciences, Nagasaki University, Nagasaki, Japan; 6 Department of Neurology and Strokology, Nagasaki University Hospital, Nagasaki, Japan

**Keywords:** Systemic sclerosis, Autoantibody, Ganglionic acetylcholine receptor, Gastrointestinal manifestations

## Abstract

**Background:**

Patients with systemic sclerosis (SSc) complicated by gastrointestinal dysmotility are difficult to treat and have high mortality. To clarify the pathogenesis of gastrointestinal manifestations, we aimed to demonstrate the association among the clinical features of SSc, the serological markers, the autoantibodies against nicotinic acetylcholine receptor at autonomic ganglia (gAChR).

**Methods:**

Fifty patients were enrolled and divided into two groups according to the presence or absence of gastrointestinal manifestations, and the characteristics were analyzed between these two groups. We measured biomarkers and the autoantibodies against two gAChRα3 and β4 subunits to test sera samples. Furthermore, patients were classified based on the presence or absence of anti-gAChR autoantibodies, and their clinical features were compared.

**Results:**

In patients with SSc and gastrointestinal manifestations, digital ulcers were more frequent (*p* = 0.050) and VEGF expression was significantly higher (*p* = 0.038). Seven subjects with SSc were seropositive for α3 subunit, whereas one patient was seropositive for β4 subunit. The mean level of anti-gAChRα3 autoantibodies in SSc patients with gastrointestinal manifestations was significantly higher than that in SSc patients without gastrointestinal manifestations (*p* = 0.001). The group of patients with SSc and gAChR autoantibodies had significantly higher endostatin levels (*p* = 0.046).

**Conclusions:**

This study is the first to demonstrate that clinical characteristics of SSc patients with seropositivity for gAChR autoantibodies. Patients with SSc have circulating autoantibodies against gAChR, which may contribute to gastrointestinal manifestations associated with this disease, suggesting that gAChR-mediated autonomic neurotransmission may provide a pathomechanism for gastrointestinal dysmotility in SSc.

## Background

Autoimmune gastrointestinal dysmotility (AGID) is recognized as a limited form of autoimmune dysautonomia [1, 2]. Gastrointestinal dysmotility in the patients with autoimmune autonomic ganglionopathy (AAG) is often prominent and may be profound, in some cases [3, 4]. Autoantibodies (Abs) targeting the ganglionic neuronal nicotinic acetylcholine receptor (gAChR) in autonomic ganglia can potentially cause widespread autonomic dysfunction, leading to AAG [5, 6]. Several studies have been performed to evaluate the frequency and specificity of serum Abs against neuronal, glial, and muscle antigens in patients clinically diagnosed with AGID, including those with achalasia, gastroparesis, pyloric stenosis, colonic inertia, and intestinal pseudo-obstruction [1, 2, 7–10]. AGID has been clinically associated with cancer and autoimmune rheumatic diseases, and the sera of patients with gut-motility disorders were screened in a few studies for antibodies specific to voltage-gated potassium channels, voltage-gated calcium channels of the P/Q- and N-type, glutamic acid decarboxylase, and neuronal gAChRα3 [7–10]. Recently, we reported that patients with AAG presenting anti-gAChR Abs had severe motility disorders [3, 4, 11], and demonstrated the existence of the gAChR Abs-related AGID in Japan [12].

Systemic sclerosis (SSc), a multi-systemic disorder of the connective tissues, is characterized by widespread vascular damage and fibrosis of the skin and visceral organs [13, 14] Gastrointestinal (GI) manifestations occur frequently in patients with SSc [15–17]. Pseudo-obstruction, malabsorption, gastroesophageal reflux disease (GERD), nausea, vomiting, constipation, and diarrhea are some of the GI complications that can occur in SSc [18–22]. In addition, autonomic dysfunction is also common in SSc, starting early during the disease process and sometimes precedes the development of fibrosis [23–27]. The pathogenesis of GI involvement is thought to include early vascular damage to the vasa nervorum [28]. This leads to neurological dysfunctions, particularly those involving autonomic pathways.

Humoral immunity dysregulation has been recognized to play an important role in SSc pathogenesis, and numerous Abs can be detected in the sera of patients with SSc [29]. Three Abs are specific for SSc and serve as specific markers. The anti-centromere antibody (ACA) is the most common marker of limited cutaneous SSc (lcSSc). Anti-scleroderma-70 (anti-Scl-70) is detected mostly in diffuse cutaneous SSc (dcSSc), although 25 to 30% of patients positive for anti-Scl-70 may have lcSSc disease. Anti-RNA polymerase III is also an important biomarker associated with severe dcSSc and a 25% risk of renal crisis [29]. Earlier studies demonstrated that GI dysmotility in SSc was associated with circulating Abs against the muscarinic AChRs and myenteric neurons [19, 29–35]. Recently, McMahan and colleagues reported patients with SSc and anti-RNPC3 antibodies had moderate-to-severe GI disease and interstitial lung disease [36].

Recently, we presented the roles of serum biomarkers and endothelial function measurement by Endo-PAT in predicting organ involvement in patients with SSc [37], and we established a luciferase-reporter immunoprecipitation system (LIPS) assay for detecting the gAChR Abs in a recent study from our laboratory. Using this method, we then aimed to confirm with two step analysis the relationship between the clinical features of SSc, especially GI manifestations, the serum biomarkers, and the anti-gAChR Abs.

## Patients and methods

### Ethical approval

All subjects provided written, informed consent to participate in this study. Ethical approval was granted by the Ethics Committees of Nagasaki Kawatana Medical Center (approval number 2011-21) and the Nagasaki University Graduate School of Biomedical Sciences (approval number 11032820).

### Patients and study design

This study was cross-sectional. Sixty-three Japanese patients with SSc, who fulfilled the 2013 classification criteria for systemic sclerosis [38], were consecutively recruited for this study from May 2011 to March 2015. The patients visited Nagasaki University Hospital and Nagasaki Kawatana Medical Center. Thirteen patients were excluded due to insufficient data. Finally, 50 patients with SSc (mean age, 62.2 ± 9.7 years; 6 males and 44 females) were analyzed. Serum and plasma were obtained from all subjects and stored. Patients were grouped according to the classification system proposed by LeRoy et al. [39]: 34 patients had lcSSc and 16 patients had dcSSc. Six patients had been treated with low-dose corticosteroids (prednisolone, < 10 mg daily). Four patients had received immunosuppressants including methotrexate, cyclosporin, and tacrolimus. No patients had received vasodilators, such as endothelin receptor antagonists and phosphodiesterase type 5 inhibitors. Ten patients treated with calcium channel blockers against hypertension and nine patients were treated with antiagrregants. All patients underwent a full medical history and physical examination. We reviewed each patient for the presence or absence of comorbid disease, Raynaud’s phenomenon, digital ulcers, GERD, renal crisis, interstitial pneumonitis, and pulmonary hypertension [34]. Laboratory assessments included blood tests, pulmonary function tests, carbon monoxide diffusion capacity testing, chest X-ray and/or high-resolution computed tomography, and transthoracic echocardiography [37].

### Clinical assessment of autonomic function and biomarker measurements

Comprehensive clinical and neurological assessments were performed for all patients [11, 40]. We reviewed clinical survey data and summaries for the patients whether they had any of the following symptoms, which would indicate dysfunction of the autonomic system: orthostatic hypotension (OH) or orthostatic intolerance (OI), arrhythmia, pupillary dysfunction, coughing episodes, dryness of the skin, hypohidrosis or anhidrosis associated with heat intolerance, appetite loss, nausea/vomiting, early satiety, postprandial abdominal pain, and gastroparesis associated with dysfunction of the upper gastrointestinal system, diarrhea or constipation, and paralytic ileus associated with dysfunction of the lower gastrointestinal system, dysuria or urinary retention associated with bladder dysfunction, and sexual dysfunction. Evidence of GI manifestation was determined by physician documentation in the clinical records and/or the presence of at least one symptom of upper and lower digestive systems described above. Gastric and enteric involvement were assessed by performing bedside patient examinations, reviewing the patients’ records, and interviewing the patients’ families. GERD was ascertained by performing a barium esophagography using the multiphasic cine technique, and esophagitis was assessed by a gastrofiberscope.

Measurements of serum biomarkers, including GDF-15 (R&D Systems, Minneapolis, MN), P*l*GF (R&D Systems), endostatin (R&D Systems), and VEGF (R&D Systems), and the plasma levels of PTX3 (Perseus Proteomics, Tokyo, Japan) were performed as described previously [37].

In the first step of the present study, patients with SSc were classified into two groups according to the presence or absence of GI manifestations and compared the clinical features and biomarkers described above.

### LIPS assay for Abs against gAChR

In the second step of the present study, serum gAChR Abs from the patients with SSc were detected by performing the LIPS assay. Subsequently, patients with SSc were classified into the two categories depending of the presence or absence of the gAChR Abs. We compared these two groups and attempted to identify the clinical characteristic of the SSc patients who were seropositive for the gAChR Abs.

We previously established and reported the use of LIPS to diagnose AAG based on IgGs against both the α3 and β4 gAChR subunits in patient serum samples [11]. We measured the gAChR Abs at the Nagasaki Kawatana Medical Center, as previously described [11]. To evaluate the diagnostic accuracy of the LIPS assay, we verified the cut-off points for all data collected in the previous study [41]. Based on the anti-gAChRα3 and β4 Abs data from the healthy control subjects, the cut-off values were calculated as the mean plus three standard deviations (SDs) from the mean [42]. In this study, antibody levels were expressed as an antibody index (AI) that was calculated as follows: AI = (measured relative luminescence units [RLU] value for the serum sample)/(the RLU cut-off value). The normal value established in this study from healthy individuals was an AI of < 1.0.

### Statistical analysis

SigmaPlot® was used for data analysis. Statistical analysis was performed to compare the prevalence of symptoms and associated data between patients with SSc and GI manifestations and those without GI manifestations. The normally distributed data in both groups of patients with SSc were analyzed by a *t* test for the continuous variables (age, age at onset, disease duration, all laboratory data, and the levels of Abs and biomarkers). The Mann–Whitney *U* test was employed in cases where the frequencies of Abs and other patient data were not normally distributed. For the categorical variables, Fisher’s exact test was used. For all analyses, *p* < 0.05 was considered to reflect a statistically significant difference.

## Results

### Detection of gAChR Abs in patients with SSc

Fourteen percent (7 of 50) of the sera samples from patients with SSc were positive for anti-gAChR Abs. More specifically, anti-gAChRα3 antibodies were detected in 7 samples, whereas anti-gAChRβ4 antibodies were detected in 1 sample (2%, 1 of 50). Further, 1 of the serum samples in the group of patients with SSc and GI manifestations was positive for both antibodies.

### Clinical features of patients with SSc with or without GI manifestations

Only one patient described below demonstrated systemic dysautonomia such as OH/OI, pupillary dysfunction, and GI manifestations. Except for this patient, no patients exhibited systemic dysautonomia. However, GI manifestations were observed in 19 patients with SSc (38%) and were composed of various digestive-system problems, such as GERD (14/19, 74%), constipation (10/19, 53%), appetite loss (9/19, 47%), vomiting (4/21, 21%), early satiety (3/19, 16%), ileus (2/19, 11%), and diarrhea (1/19, 5%). Tables 1 and 2 summarize the clinical profiles, SSc Abs, laboratory data, serum biomarkers, and gAChR Abs for patients with SSc, either with or without GI manifestations. No significant differences in the clinical profile, SSc Abs, and laboratory data were noted. Various autonomic dysfunction was confirmed in one seropositive case only (patient 1 in Table 2).

**Table 1:** Comparison of the clinical features of SSc patients, with and without GI manifestations

	SSc with GI manifestations (*n* = 19)	SSc without GI manifestations (*n* = 31)	*p* value
Age (years)	62.1 ± 8.0	62.3 ± 10.7	0.934
Age at onset (years)	54.9 ± 11.5	55.3 ± 12.9	0.913
Disease duration (years)	7.2 ± 7.5	7.0 ± 8.8	0.602
Sex, female (%)	18 (94.7)	26 (83.9)	0.834
Diffuse SSc (%)	9 (47.4)	8 (25.8)	0.394
Raynaud’s phenomenon (%)	19 (100.0)	31 (100.0)	1.000
Digital ulcers (%)	6 (31.6)	3 (9.7)	0.149
Renal crisis (%)	2 (10.5)	0 (0.0)	0.158
Interstitial pneumonitis (%)	8 (42.1)	12 (38.7)	1.000
Pulmonary hypertension (%)	2 (10.5)	1 (3.2)	0.555

*Abbreviations*: *GI* gastrointestinal manifestations, *SSc* systemic sclerosis

**Table 2:** Summary of the characteristics of 19 patients with SSc and GI manifestations

Patient no. (years, sex)	SSc duration, years	Anti-gAChR Abs	Levels of anti-gAChR Abs ^a^, α3/β4	Other Abs	Complications	Type of SSc	Clinical features (RP, DU, RC, IP, PH)	GI manifestation (GERD, PI, C, D, and other symptoms ^b^)
1 (76, F)	3	Positive (α3)	3.607 (+)/0.212 (−)	Scl-70	RA	Diffuse	RP, DU, IP	C, D
2 (57, F)	4	Positive (α3, β4)	2.694 (+)/1.766 (+)	ACA		Limited	RP	GERD
3 (64, F)	12	Positive (α3)	1.200 (+)/0.516 (−)	RNP	SS	Diffuse	RP, PH	GERD
4 (63, M)	0	Positive (α3)	1.025 (+)/0.838 (−)	(Negative)	PM	Diffuse	RP, RC, IP	GERD, appetite loss, nausea/vomiting, early satiety, PI, C
5 (57, F)	3	Negative	0.420 (−)/0.248 (−)	ACA	SS, PBC	Limited	RP	GERD, C
6 (75, F)	2	Negative	0.431 (−)/0.272 (−)	Scl-70	SS	Diffuse	RP, DU, RC, IP	Appetite loss
7 (64, F)	0	Negative	0.817 (−)/0.482 (−)	ACA	Chronic thyroiditis	Limited	RP, IP	GERD
8 (63, F)	26	Negative	0.368 (−)/0.207 (−)	ARS		Limited	RP, IP	Appetite loss, C
9 (61, F)	22	Negative	0.499 (−)/0.582 (−)	Scl-70		Diffuse	RP, DU, IP	GERD, appetite loss, C
10 (63, F)	18	Negative	0.354 (−)/0.209 (−)	Scl-70	GPA, RA	Diffuse	RP, IP	GERD, appetite loss, nausea/vomiting, C
11 (49, F)	10	Negative	0.306 (−)/0.325 (−)	ACA	SS	Limited	RP	GERD, appetite loss
12 (66, F)	4	Negative	0.309 (−)/0.242 (−)	ACA		Limited	RP	C
13 (44, F)	7	Negative	0.350 (−)/0.255 (−)	Scl-70	Chronic thyroiditis	Diffuse	RP, DU	GERD
14 (68, F)	3	Negative	0.271 (−)/0.277 (−)	ACA		Limited	RP	GERD
15 (66, F)	10	Negative	0.436 (−)/0.420 (−)	Scl-70		Diffuse	RP, DU, IP	GERD, appetite loss
16 (63, F)	2	Negative	0.416 (−)/0.253 (−)	ACA		Limited	RP	Appetite loss, nausea/vomiting, early satiety, C
17 (69, F)	6	Negative	0.540 (−)/0.355 (−)	(Negative)		Limited	RP, DU, PH	GERD
18 (51, F)	2	Negative	0.334 (−)/0.336 (−)	ACA	Chronic thyroiditis	Limited	RP	GERD, C
19 (60, F)	2	Negative	0.271 (−)/0.253 (−)	(Negative)		Diffuse	RP	GERD, appetite loss, nausea/ vomiting, early satiety, C

*Abbreviations*: *Abs* autoantibodies, *ACA* anti-centromere antibodies, *ARS* anti-aminoacyl-tRNA synthetase antibodies, *C* constipation, *D* diarrhea, *DU* digital ulcers, *F* female, *gAChR* ganglionic acetylcholine receptor, *GERD* gastroesophageal reflux disease, *GI* gastrointestinal, *GPA* granulomatosis with polyangiitis, *IP* interstitial pneumonitis, *M* male, *PBC* primary biliary cirrhosis, *PH* pulmonary hypertension, *PI* paralytic ileus, *PM* polymyositis, *RA* rheumatoid arthritis, *RC* renal crisis, *RP* Raynaud’s phenomenon, *SS* Sjögren’s syndrome, *SSc* systemic sclerosis

^a^The normal value of gAChR Abs established from healthy individuals was < 1.0 AI

^b^Other GI manifestations of symptoms included appetite loss, nausea/vomiting, early satiety, and postprandial abdominal pain associated with dysfunction of the upper digestive system

**Fig. 1:**
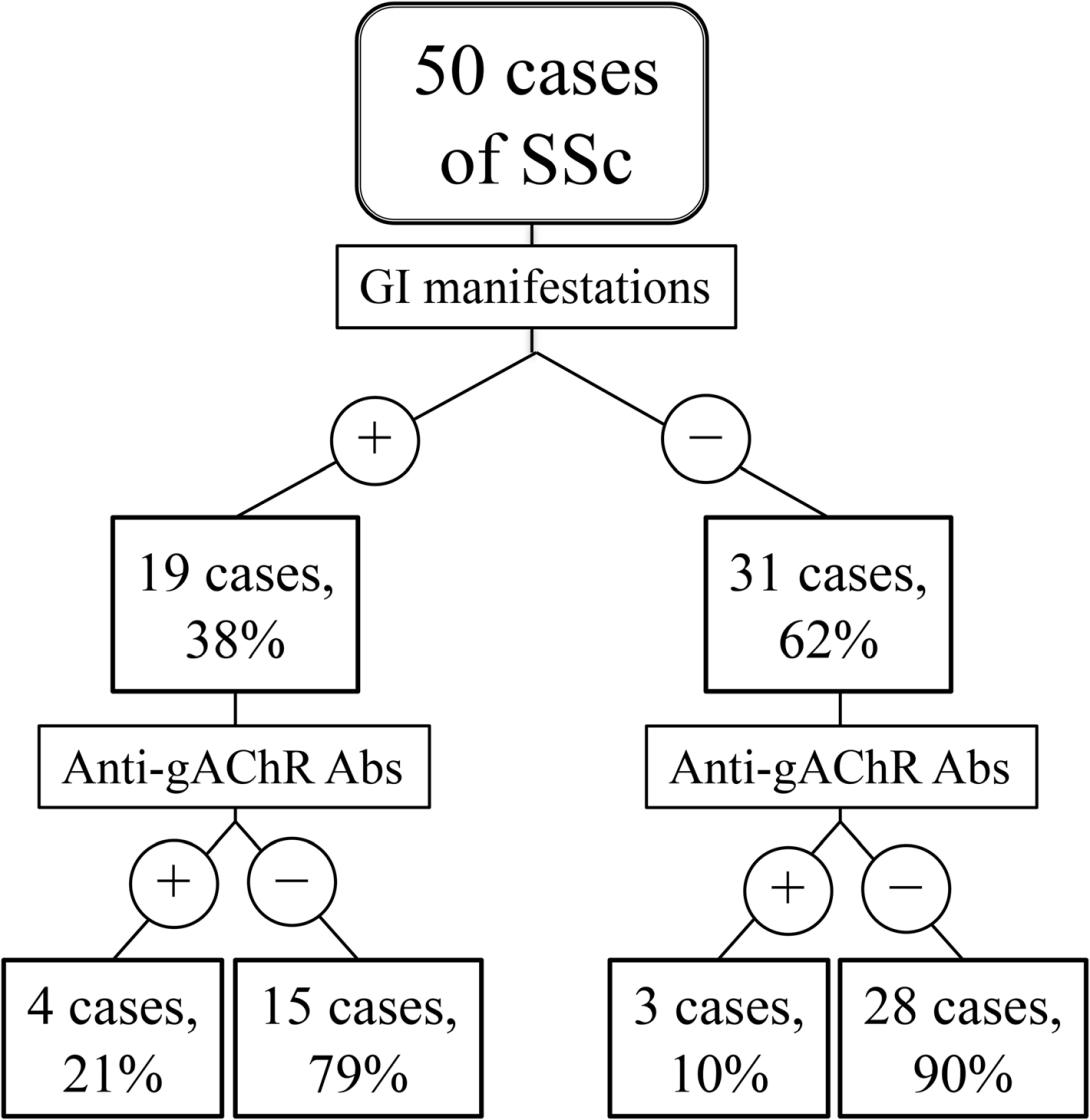
Schematic representation of study design. Details regarding study design and recruitment for patients in each group. We tested sera from patients with SSc. Using the LIPS assay, we detected autoantibodies against gAChR in 21% (4 of 19) of samples from patients with SSc and GI manifestations, and in 10% (3 of 31) of samples from patients with patients without GI manifestations

The respective mean levels of anti-gAChRα3 Abs in patients with GI manifestations (+) and GI manifestations (−) were 0.771 AI and 0.452 AI (*p* = 0.001; Table 3), and the respective mean levels of anti-gAChRβ4 Abs in patients with GI manifestations (+) and GI manifestations (−) were 0.424 AI and 0.316 AI (*p* = 0.318; Table 3). The frequencies of gAChR Abs in the groups of SSc patients with GI manifestations (+) or without GI manifestations (−) were 21% (4 of 19), and 10% (3 of 28), respectively (*p* = 0.273; Fig. 1 and Table 3).

**Table 3:** Comparison of laboratory findings between SSc patients with and without GI manifestations

	SSc with GI manifestations (*n* = 19)	SSc without GI manifestations (*n* = 31)	*p* value
Seropositive for Anti-gAChRα3 Abs (%)	4 (21.1)	3 (9.7)	0.273
Seropositive for Anti-gAChRβ4 Abs (%)	1 (5.3)	0 (0.0)	0.216
Anti-gAChRα3 Abs (A.I.)	**0.771 ± 0.889**	**0.452 ± 0.481**	0.001*
Anti-gAChRβ4 Abs (A.I.)	0.424 ± 0.363	0.316 ± 0.160	0.318
Other Abs			
ACA (+) (%)	8 (42.1)	16 (51.6)	0.799
Anti-Scl70 Abs (+) (%)	6 (31.6)	6 (19.4)	0.521
Other Abs (+) (%)	2 (10.5)	4 (12.9)	1.000
Negative (%)	3 (15.8)	6 (16.1)	1.000
Other laboratory data			
IgG (mg/dL)	1641.9 ± 440.9	1536.4 ± 371.5	0.399
KL-6 (U/mL)	624.0 ± 692.3	560.2 ± 649.5	0.699
NT-proBNP (pg/mL)	211.5 ± 372.7	202.7 ± 273.0	0.757
%FVC	101.2 ± 20.6	99.4 ± 24.5	0.805
FEV1.0%	83.1 ± 9.1	78.0 ± 17.2	0.120
%DLCO	70.5 ± 21.9	70.6 ± 17.2	0.992
DLCO/VA	81.0 ± 19.8	85.5 ± 16.3	0.427
TR-PG	26.7 ± 9.9	23.3 ± 9.3	0.259
Estimated PA systolic pressure	30.1 ± 6.4	28.4 ± 9.3	0.539
EF	71.6 ± 6.5	70.6 ± 6.0	0.611
Serum biomarkers			
VEGF (pg/mL)	**642.4 ± 510.5**	**389.6 ± 423.0**	0.038*
PIGF (pg/mL)	15.7 ± 4.8	14.7 ± 6.3	0.270
GDF-15 (pg/mL)	1482.4 ± 906.9	1422.2 ± 947.0	0.366
PTX3 (ng/mL)	3.3 ± 1.7	2.8 ± 1.0	0.414
Endostatin (ng/mL)	85.3 ± 20.2	85.4 ± 20.0	0.990
TGFβ1	10,580.8 ± 7998.2	14,707.5 ± 8058.7	0.060

*p < 0.05 was considered statistically significant

*Abbreviations*: *Abs* autoantibodies, *ACA* anti-centromere antibodies, *DLCO* diffusing capacity of lung for carbon monoxide, *EF* ejection fraction, *FEV* forced expiratory volume, *FVC* forced vital capacity, *gAChR* ganglionic acetylcholine receptor, *GDF-15* growth differentiation factor-15, *GI* gastrointestinal, *PA* pulmonary artery, *PIGF* placenta growth factor, *PTX3* pentraxin 3, *SSc* systemic sclerosis, *TGFβ1* transforming growth factor β1, *TR-PG* tricuspid regurgitation pressure gradient, *VEGF* vascular endothelial growth factor

Analysis of serum biomarkers revealed that VEGF production was significantly higher in the group of patients with SSc and GI manifestations than in the group with SSc that lacked GI manifestations (*p* = 0.038; Table 3). No correlations were found between the presence of gAChR Abs and clinical profiles, SSc Abs, laboratory data, and serum biomarkers during statistical analysis with all of the samples.

### Clinical features of patients with SSc and gAChR Abs

The detailed clinical features of patients with SSc, with or without anti-gAChR Abs, are shown in Table 4. In 7 patients with SSc and gAChR Abs, 3 patients (42.9%) had GERD, 2 patients (28.6%) had constipation, 1 patient (14.3%) had paralytic ileus, and 1 patient (14.3%) had diarrhea (Table 4), with some overlap in symptoms occurring between patients. In contrast, the frequencies of each GI manifestation were low in patients with SSc who did not have gAChR Abs, as compared to patients with SSc and gAChR Abs. A tendency was observed where the clinical symptoms of patients with SSc, such as digital ulcers, renal crisis, interstitial pneumonitis, and pulmonary hypertension, were more frequent in patients with SSc that also had gAChR Abs (Table 4). Moreover, while analyzing serum biomarkers in this study, we found that endostatin levels were significantly higher in patients with SSc and gAChR Abs than in SSc patients without gAChR Abs (*p* = 0.046; Table 4).

**Table 4:** Clinical profiles of patients with SSc in the presence or absence of gAChR Abs

Characteristics	SSc with gAChR Abs (*n* = 7)	SSc without gAChR Abs (*n* = 43)	*p* value
Age (years)	62.9 ± 14.0	62.1 ± 9.0	0.764
Age at onset (years)	57.1 ± 12.5	54.8 ± 12.4	0.646
Disease duration (years)	5.7 ± 5.6	7.3 ± 8.7	0.911
Sex, female (%)	6 (85.7)	38 (88.4)	1.000
Diffuse SSc (%)	4 (57.1)	13 (30.2)	0.451
Raynaud’s phenomenon (%)	7 (100.0)	43 (100.0)	1.000
Digital ulcers (%)	2 (28.6)	7 (16.3)	0.615
Renal crisis (%)	1 (14.3)	1 (2.3)	0.287
Interstitial pneumonitis (%)	4 (57.1)	16 (37.2)	0.717
Pulmonary hypertension (%)	1 (14.3)	2 (4.7)	0.394
GI manifestations			
GERD (%)	3 (42.9)	11 (25.6)	0.677
Paralytic ileus (%)	1 (14.3)	1 (2.3)	0.287
Constipation (%)	2 (28.6)	8 (18.6)	0.637
Diarrhea (%)	1 (14.3)	0 (0.0)	0.157
Other Abs			
ACA (+) (%)	3 (42.9)	22 (51.2)	1.000
Anti-Scl70 Abs (+) (%)	2 (28.6)	10 (23.3)	1.000
Other Abs (+) (%)	1 (14.3)	4 (9.3)	0.559
Negative (%)	1 (14.3)	7 (16.3)	1.000
Other laboratory data			
IgG (mg/dL)	1775.0 ± 614.2	1548.8 ± 363.6	0.459
KL-6 (U/mL)	666.0 ± 678.3	572.6 ± 642.4	0.534
NT-proBNP (pg/mL)	114.1 ± 128.9	217.4 ± 418.6	0.530
%FVC	109.8 ± 25.5	98.8 ± 22.3	0.311
FEV1.0%	78.5 ± 13.6	79.9 ± 10.0	0.786
%DLCO	61.9 ± 23.8	71.7 ± 17.5	0.262
DLCO/VA	70.5 ± 15.6	85.7 ± 17.2	0.068
TR-PG	28.4 ± 17.1	24.0 ± 7.9	0.953
Estimated PA systolic pressure	26.5 ± 8.5	29.3 ± 8.4	0.528
EF	71.6 ± 3.4	70.8 ± 6.7	0.800
Serum biomarkers			
VEGF (pg/mL)	441.6 ± 112.1	480.2 ± 476.5	0.631
PIGF (pg/mL)	16.6 ± 6.6	14.8 ± 8.6	0.482
GDF-15 (pg/mL)	1595.9 ± 1529.0	1423.1 ± 1086.5	0.941
PTX3 (ng/mL)	2.3 ± 1.2	3.1 ± 1.5	0.336
Endostatin (ng/mL)	**101.8 ± 21.4**	**83.3 ± 18.7**	0.046*
TGFβ1	13,038.6 ± 12,382.6	13,334.3 ± 7692.2	0.506
Immunotherapies			
Oral PSL (%)	1 (14.3)	6 (14.0)	1.000
Immunosuppressants (%)	1 (14.3)	3 (7.0)	0.532
Steroid pulse therapy (%)	0 (0.0)	0 (0.0)	1.000
IVIg (%)	0 (0.0)	0 (0.0)	1.000
Plasmapheresis (%)	0 (0.0)	0 (0.0)	1.000

*p < 0.05 was considered statistically significant

*Abbreviations*: *Abs* autoantibodies, *ACA* anti-centromere antibodies, *DLCO* diffusing capacity of lung for carbon monoxide, *EF* ejection fraction, *FEV* forced expiratory volume, *FVC* forced vital capacity, *gAChR* ganglionic acetylcholine receptor, *GDF-15* growth differentiation factor-15, *GERD* gastroesophageal reflux disease, *GI* gastrointestinal, *PA* pulmonary artery, *PIGF* placenta growth factor, *PTX3* pentraxin 3, *SSc* systemic sclerosis, *TGFβ1* transforming growth factor β1, *TR-PG* tricuspid regurgitation pressure gradient, *VEGF* vascular endothelial growth factor

## Discussion

This study is the first to our knowledge to describe the clinical characteristics and biomarkers of the SSc patients who were positive for gAChR Abs. In this study, autonomic function and humoral autoimmunity against the autonomic nervous system were investigated in patients affected by SSc, with a particular focus on GI dysmotility in those patients. Here, we report four major findings: (i) we determined the frequencies of Abs against gAChR in SSc patients with GI manifestations, (ii) the mean levels of anti-gAChRα3 Abs and VEGF were significantly higher in the SSc with GI manifestations group than in the SSc without GI manifestations group, and (iii) endostatin was significantly higher in SSc patients with gAChR Abs than in SSc patients without gAChR Abs.

SSc is a chronic autoimmune disease, and the most common clinical presentations include Raynaud’s phenomenon, skin thickening, and tightness caused by widespread vasculopathy and excessive fibrosis [13]. The GI tract is the commonly involved internal organ in SSc. Vascular changes, collagen accumulation in the submucosa, and smooth muscle atrophy are histological hallmarks of SSc found in the digestive walls of patient biopsies and autopsies [16, 20, 21]. However, recently, the association between Abs and SSc GI damage has attracted great interest. We found that 21% of the SSc patients with GI manifestations were seropositive for anti-gAChR Abs in this study. The gAChR Abs can potentially impair autonomic ganglionic synaptic transmission. Additionally, because both the sympathetic and parasympathetic ganglia utilize nicotinic cholinergic synapses, Abs that interfere with ganglionic transmission may cause dysautonomia [6, 43]. Furthermore, the levels of anti-gAChRα3 Abs were significantly higher in SSc patients with GI manifestations. Hence, the gAChR Abs were considered as possible pathogenic humoral factor in the pathomechanism of GI dysmotility in SSc along with Abs against M3R, RNPC3, U1 snRNP, U3 snRNP, signal recognition particle, Ku, and myenteric neuron [29, 30, 44].

Several previous studies were performed to evaluate autonomic functions in patients with SSc and identify associations with the severity of microvascular damage [13, 15]. Gigante and colleagues evaluated correlation between autonomic dysfunctions and VEGF production, and they concluded that parasympathetic dysfunction was linked to digital microvascular damage based on data related to heart rate variability and VEGF release [45]. Neuropilin 1 is a non-tyrosine kinase receptor for VEGF that plays an essential role in GI motility [46]. In the future, it will be important to verify the role of VEGF and its receptor in autonomic function, especially GI contractility and motility. In this manner, the role of VEGF in the autonomic dysfunctions in SSc could become clear.

In SSc patients with gAChR Abs, clinical profiles involving digital ulcers, renal crisis, interstitial pneumonitis, and pulmonary hypertension were more frequent, although no statistical significances were observed. Previously, some research groups investigated potential biomarkers of SSc, including endostatin, to correlate their levels with serological and clinical parameters [47]. These previous reports supported the conclusion that higher serum levels of endostatin correlated with angiogenesis and fibrosis disturbances and, thus, may play an important role in SSc. Our present data also showed a high serum concentration of endostatin in SSc patients with gAChR Abs but no clear association of endostatin with the levels of gAChR Abs. The larger sample numbers with prospective observations are necessary to reveal the role of endostatin in the pathomechanism of SSc. The relationship between the role of endostatin and gAChR Abs associated with the autonomic dysfunction has yet to be verified. However, serum endostatin levels could be related to clinical profiles described above for SSc patients with gAChR Abs [37].

Based on the facts of the present study, the clinical profiles of the group of SSc patients with GI manifestations and the group of SSc patients with the gAChR Abs did not overlap completely (Tables 1, 2, 3, and 4). Only one patient was seronegative in the group of the SSc with gAChR Abs in Table 4. Accordingly, we considered the possibility that the gAChR Abs were produced from the mechanism of SSc autoimmunity. The detailed pathogenesis of such a condition has not been fully elucidated, although GI dysmotility disorders are likely to exist within the broad clinical spectrum of AAG that is etiologically characterized by anti-gAChR Abs. For many years, SSc has been considered a manifestation of an underlying motility disorder. In 1972, Cohen et al. proposed that immune-mediated enteric dysfunction occurred in SSc [48]. This raises the possibility that motility changes may result from dysfunctions of neurons involved in the enteric nervous system, the vasculature, or elsewhere [19–21]. It is also possible that SSc GI damage occurring in such a case is a variation of AGID, considering that the anti-gAChR Abs might mediate autonomic dysfunction, contributing to the autoimmune mechanisms underlying these GI motility disorders [1, 2, 12, 17]. Several reports have shown the occurrence of anti-M3R Abs correlating with GI dysmotility in SSc [32–34], as well as the efficacy of intravenous immunoglobulin (IVIg) due to anti-idiotypic neutralization [34]. As with the gAChR, the M3R exists in human autonomic nervous system. Although GI dysmotility in SSc with the anti-gAChR Abs could be considered a form of AGID in a broad sense, we recommend future experimentation be performed to test the hypothesis that immune cell–endothelial nerve interactions (even with anti-gAChR Abs) are related autonomic dysfunction, including GI manifestations, in SSc. Our results suggest rheumatologists and neurologists should be aware of the necessity of measuring the levels of anti-gAChR Abs in SSc cases with autonomic dysfunction including GI manifestations.

## Conclusions

In conclusion, we determined the clinical characteristics of SSc patients with seropositivity for the anti-gAChR Abs, and considered the relationship among gAChR Abs, several biomarkers, and GI manifestations in SSc. However, the present study has important limitations that should be noted, namely, the observational study design and the small sample size. A prospective, clinical interventional, multicenter cohort study will be necessary to confirm the relationships between the levels of anti-gAChR Abs, serum biomarkers (including VEGF and endostatin), and autonomic function (including the severity of GI dysmotility in SSc) versus the outcomes of immunotherapies. Moreover, it is necessary to clarify whether a direct link exists between the presence of gAChR Abs and the specific interference in synaptic transmission in autonomic ganglia including GI tract.

## Data Availability

All data generated or analyzed during this study are included in this published article. Shunya Nakane had full access to all of the data in the study and takes responsibility for the integrity of the data and the accuracy of the data analysis.

## Abbreviations

AAG: Autoimmune autonomic ganglionopathy
Abs: Antibodies
ACA: Anti-centromere antibody
AGID: Autoimmune gastrointestinal dysmotility
AI: Antibody index
CVRR: Coefficient of variation in R-R intervals
dcSSc: Diffuse cutaneous SSc
gAChR: Ganglionic acetylcholine receptor
GERD: Gastroesophageal reflux disease
GI: Gastrointestinal
IVMP: Intravenous methylprednisolone
lcSSc: Limited cutaneous SSc
LIPS: Luciferase immunoprecipitation system
NE: Norepinephrine
OH: Orthostatic hypotension
OI: Orthostatic intolerance
RLU: Relative luminescence units
SD: Standard deviation
SSc: Systemic sclerosis
